# “Saying ‘I'm not okay’ is extremely risky”: Postpartum mental health, delayed help‐seeking, and fears of the child welfare system among queer parents

**DOI:** 10.1111/famp.13032

**Published:** 2024-06-23

**Authors:** Abbie E. Goldberg, Reihonna L. Frost

**Affiliations:** ^1^ Clark University Worcester Massachusetts USA; ^2^ Bridgewater State University Bridgewater Massachusetts USA

**Keywords:** bisexual, child welfare, LGBTQ+ parents, mental health, nonbinary, postpartum, transgender

## Abstract

Parent mental health challenges in the postpartum and early parenthood have profound implications for parent, child, and family well‐being. Little research has focused on postpartum mental health challenges and barriers to help‐seeking among queer birthing people, including members of this community who may be particularly vulnerable to mental health difficulties, such as queer cis women partnered with men, trans/nonbinary parents, and queer parents who are young, low‐income, and/or of color. This mixed‐methods study of queer parents (*n =* 99), all of whom were assigned female at birth (AFAB) and gave birth to a child within the past several years, explores parents' postpartum mental health difficulties and perceived barriers to seeking help. Using a structural stigma framework, this study found that participants reported high rates of postpartum mental health difficulties (89%) and reported various barriers to seeking support including fears of discrimination and being deemed “unfit” by providers, which might lead to child welfare system involvement. Young parents and low‐income parents were particularly fearful of child welfare system contact and potential child removal. Factors that encouraged help‐seeking (e.g., desire to be a good parent; partner pressure to seek help) and implications for family practitioners are discussed.

## INTRODUCTION

New parents' mental health has profound impacts on their parenting, and, by extension, child development (Netsi et al., 2018). Some parents have been largely neglected in the literature on the transition to parenthood, mental health, and family outcomes (Goldberg, 2023). Queer/bisexual women, especially those partnered with men, may be at greater risk for mental health challenges, including during the perinatal period (Flanders et al., 2016; Goldberg et al., 2020; Kirubarajan et al., 2022). Trans/nonbinary (TNB) birthing people may also face unique mental health challenges, insomuch as dysphoria, stigma, and isolation represent common themes in the nascent literature on TNB people's pregnancy and birthing experiences (Greenfield & Darwin, 2021; Hoffkling et al., 2017). Amidst societal discrimination (e.g., beliefs that LGBTQ+ people should not be parents and are inferior parents; Goldberg et al., 2024), these groups may be reluctant to seek help for mental health issues, especially in the context of other marginalized identities (e.g., due to race, income, age, or relationship status).

This study sought to understand the mental health experiences and concerns of queer parents who gave birth in the past 5 years. We were interested in how participants' experiences of postpartum mental health challenges and help‐seeking choices were impacted by concerns about stigma from health providers or social service agencies (e.g., child welfare services) related to their various intersecting identities (e.g., gender identity, sexual orientation, race, class). We aimed to enhance understanding of the multilayered concerns of a vulnerable but hidden population—a topic of significance given the serious consequences of postpartum mental health disorders on children (Netsi et al., 2018) alongside the very real barriers to help‐seeking in this marginalized group (e.g., due to concerns about structural stigma; Polikoff, 2018).

### Rationale for the current study

Several scholars (Goldberg et al., 2024; Polikoff, 2018) have pointed to the invisibility of various types of multiply marginalized (i.e., vulnerable to societal disenfranchisement) queer mothers. Of note is that, in this paper, we use “queer” to encompass lesbian, gay, bisexual, pansexual, queer, asexual, and other sexual minority identities. Most research on queer mothers focuses on White, middle‐class lesbian mothers (Goldberg et al., 2024; Lavender‐Stott & Tasker, 2020), with little attention paid to queer mothers of color, working‐class and poor queer mothers, bisexual/nonmonosexual mothers, and queer AFAB parents of diverse gender identities (Goldberg, 2023). Polikoff (2018) has emphasized the invisibility of low‐income and racial/ethnic minority queer mothers in the literature, noting their particular risk for system involvement—including criminal legal and child welfare services—and thus the importance of understanding their unique needs and experiences. Further, Goldberg et al. (2024) highlight the limited research on difficult topics pertaining to queer mothers, such as mental illness, substance abuse, and poverty. This paucity is somewhat understandable amidst the intersecting systemic biases against queer parents and the historic reluctance of researchers to focus on issues that could be used to further harm or discredit them (Goldberg et al., 2024). However, failing to study marginalized populations further erases them (Polikoff, 2018). Research is needed that contextualizes the challenges faced by vulnerable members of the LGBTQ+ community, in order to better support them.

### Research questions

Our primary research question is: How do queer parents of young children, all of whom gave birth in the past several years, describe their postpartum mental health and help‐seeking? In particular:

1. What types of mental health challenges do queer birthing parents describe postpartum?
  - What do they attribute those challenges to?
2. What barriers do these parents perceive in seeking help?
  - (How) do such barriers reflect parents' concerns about various stigmas (e.g., related to mental health, LGBTQ+ identities, race, class), which are embedded in heteronormative, racialized, and classed ideas of family (Allen & Mendez, 2018)?
  - To what extent and in what ways do parents possess a fear of child protective services specifically—a system that may be especially discriminatory toward queer mothers?

### Postpartum and early parenthood: A time of mental health challenges

As noted, general work on queer mothers has focused on White, middle‐class, middle‐aged lesbian mothers, with limited attention to the experiences of queer mothers who are young, of color, low‐income, or nonmonosexual (e.g., queer, bisexual; Goldberg, 2023). This oversight is notable because the queer community is racially diverse, and characterized by high levels of poverty, particularly among parents (Badgett et al., 2019; Gates, 2015). The focus on White queer mothers encompasses work on mental health, including the postpartum period (Goldberg & Smith, 2011; Ross & Goldberg, 2016), a time of increased stress and mental health challenges for birthing parents in particular (Neely et al., 2023). The prevalence of perinatal mental health problems in the general population ranges from 12%–35%, with notable variation depending on individuals' social locations (Neely et al., 2023). Socioeconomic disadvantages, relationship difficulties/violence, low social support, and negative birthing experiences combine with the stresses of pregnancy and new parenthood to increase a birthing person's risk for mental health challenges (Lafferty et al., 2023; Neely et al., 2023).

Although research on queer women's postpartum mental health is limited, existing work suggests that they report higher rates of postpartum mental health challenges than heterosexual mothers, which may be related to experiences of stigma and exclusion from prenatal care (Flanders et al., 2016; Kirubarajan et al., 2022). Further, research points to subgroups of queer parents who may be at greater risk for mental health problems, including in the perinatal period. Preliminary evidence suggests that TNB birthing parents face unique perinatal vulnerabilities, including traumatic birth and postpartum mental health difficulties, in part due to the cissexism and erasure they face in health care settings (Greenfield & Darwin, 2021). Further, bisexual/queer women partnered with men have shown poorer mental, sexual, and reproductive health outcomes than female‐partnered LBQ+ women (Dyar et al., 2014; Flanders et al., 2016; Januwalla et al., 2019). Such health disparities have been attributed to bisexual women's exposure to bisexual stigma (e.g., stereotypes of bisexual women as hypersexual and unstable; Bostwick, 2012; Ross et al., 2017) and bisexual invisibility (e.g., being “read” as heterosexual or lesbian; Flanders et al., 2016). Health disparities may also reflect disparities in income: bisexual women are more likely to be living in poverty than other segments of the LGBTQ+ community (29.4%), with the exception of trans adults (29.4%); such numbers compare to 19.5% for bisexual cis men, 17.9% for lesbian cis women, and 12.1% for gay cis men (Badgett et al., 2019).

According to an intersectional framework (Allen & Mendez, 2018), LGBTQ+ parents who possess additional marginalized statuses may experience greater burdens to their mental health, which combine with the stigma that parents face in seeking or receiving treatment (Hsieh & Shuster, 2021). For example, single parents are at risk for postpartum mental health issues—risk that is largely accounted for by economic factors (Crosier et al., 2007) as parents in poverty are also at risk for postpartum mental health problems (Goyal et al., 2010). Postpartum mental health issues are also elevated for Black birthing people (James et al., 2023) and other racial/ethnic minorities (e.g., Indigenous women; Owais et al., 2020), who are less likely to seek out and receive appropriate mental health treatment due to barriers to care (e.g., racialized stigmas).

### Help‐seeking experiences, concerns, and barriers

Women in general cite a variety of barriers to seeking help for postpartum depression, anxiety, and other perinatal mental health challenges. These include financial challenges, accessibility issues (e.g., limited provider availability), prior negative experiences with health providers, discouragement from seeking treatment by family/partner, and stigmas associated with mental health issues and help‐seeking, including internalized stigmas (e.g., I am weak/a failure if I am depressed/seek help; Dennis & Chung‐Lee, 2006; Hadfield & Wittkowski, 2017; Hansotte et al., 2017). In that parents with mental illness are at higher risk for child welfare involvement and custody loss than parents without mental illness (Kaplan et al., 2019), parents experiencing mental health challenges may be reluctant to disclose such issues to providers in part because of fears of child protective services [CPS] involvement (i.e., “being reported” to CPS; Forder et al., 2020; Johnson et al., 2023). Using a mixed‐methods approach, Forder et al. (2020) studied women in Australia and found that 21% of women reported not having always responded honestly about their mental health in the perinatal period, citing stigma‐related concerns and fear of adverse consequences, including involvement of social services, as reasons for not being honest. Notably, women who were most likely to report perinatal mental health challenges were also the least likely to be open about their mental health (Forder et al., 2020).

Concerns about stigma may be amplified among queer/bisexual new parents with mental health issues, as LGBTQ+ people face systematic biases in the health care system (Hsieh & Shuster, 2021; Sabin et al., 2015). Not only might such parents fear biased treatment, but they may worry that provider biases could lead them to deem them “unfit” due to the intersection of their mental health issues and sexual orientation/gender identity, rendering them vulnerable to CPS referral. Queer mothers, and mothers who are young, low‐income, and of color, may be overrepresented in the child welfare system in part due to systemic biases (Harp & Oser, 2016). Indeed, the child welfare system is sometimes critically referred to as the family regulation system (Polikoff & Spinak, 2021) to emphasize the ways that it often polices, controls, and interferes with families rather than providing support or positively impacting families' welfare. Systemic bias operates, in part, through the coding of poverty as neglect: in turn, poor parents are often presumed to be less competent as parents, and therefore in need of supervision or control rather than resources or support (Fong, 2017; Polikoff & Spinak, 2021). Social worker bias, greater social services contact for low‐income families, and policy wording that can result in differential enforcement based on class and race may contribute to the overrepresentation of low‐income and Black and Indigenous mothers in particular in the child welfare system (Cénat et al., 2021; Fong, 2017).

#### Fear of social service systems

Fear of social services, then, may be elevated among certain vulnerable groups. Concerns about being reported to child welfare systems may be heightened among poor mothers, who seek to avoid situations where their parenting might be scrutinized (e.g., such as in a medical context) and may downplay their mental health issues when faced with direct questions from health providers (Fong, 2017). LGBTQ+ parents also recognize their vulnerability to having their identities scrutinized by such systems and may fear them (Washington, 2022). LGBTQ+ parents are sensitive to the reality that child welfare services, as a government system, reflect larger societal values that treat heterosexual, cis, and two‐parent families as ideal and normative (Washington, 2022). Indeed, a study of over 600 Black mothers in the U.S., 21% of whom were lesbian/bisexual, found that lesbian/bisexual mothers were more than four times likely to have lost custody of their children to the child welfare system than heterosexual mothers (Harp & Oser, 2016). The authors suggest that this may reflect system‐level bias (i.e., tendency to label certain mothers “unfit”) or other factors related to women's sexual minority status (e.g., lower income). A study of stigmatized experiences among 39 adults raised by LGBTQ+ parents found that five reported childhood poverty as a stigmatized experience; of these, four felt the need to hide their economic status for fear of child welfare interference (Goldberg et al., 2024).

Concerns about the child welfare system may also be heightened among queer parents who are not only low income but also young. Kuri (2020) explored the experiences of young (e.g., teen/early 20s), often financially challenged, queer mothers, a group that has historically been absent from LGBTQ+ parenting literature. This study describes how these women experienced multiple types and layers of social exclusion, not only from heterosexual married suburban moms but also middle‐aged, middle‐class, lesbian mother communities, with whom they do not share the stigma and stressors of poverty or teen parenthood (Kuri, 2020). Although not specifically about child welfare involvement or related fears, this study points to the scrutiny and moral judgment experienced by this multiply marginalized group of queer young mothers.

LGBTQ+ parents' concerns about scrutiny may be heightened in the current sociopolitical climate (Washington, 2022). Numerous anti‐LGBTQ+ bills have been proposed or passed in states across the U.S., and parents have been threatened with child welfare interference if their parenting is deemed to threaten sexual and gender norms (Washington, 2022). A recent study by Radis and Nadan (2021) of 15 Black lesbian mothers highlighted how parents of multiple marginalized statuses experienced heightened fears about their family's safety amidst the intersectional risks posed by the Trump presidency. The increased visibility and power of white supremacy culture and the conservative evangelical movement created a climate where women felt increasingly worried about their rights. This study highlights the vulnerability that Black queer mothers specifically may experience related to their unique intersecting statuses.

In addition to increased scrutiny, the divisiveness of the current political climate and normalization of bias toward minoritized individuals may increase the isolation of LGBTQ+ families from their broader communities and families. In a study of LG adoptive parents after the 2016 election, Gabriele‐Black et al. (2021) found that parents, who largely experienced strong and negative reactions to the election of President Trump, often took actions to decrease the visibility of their families' queer nature in schools and public settings for fear of discrimination. Participants also described conflict with extended family due to differences in political beliefs and voting decisions, leading to distancing from previously supportive relationships. This study reveals how the sociopolitical climate can impact the mental health and available social supports of queer parents—which is especially notable given the significance of social support as a protective factor for new parents, whereas judgmental or conflictual social relationships can worsen postpartum mental health outcomes (Ross & Goldberg, 2016; Sun & Mulvaney, 2021).

### Theoretical framework

This study is guided by a structural stigma framework, which defines structural stigma as a construct that includes “societal‐level conditions, cultural norms, and institutional policies that constrain the opportunities, resources, and well‐being of the stigmatized” (Hatzenbuehler & Link, 2014, p. 2). In this study, structural stigma encompasses both the health care and child welfare systems. Indeed, prior research has established how structurally stigmatizing conditions embedded in both health care (Falck & Bränström, 2023) and social service systems (McGrath et al., 2023) contribute to and perpetuate negative outcomes, including system mistrust, in vulnerable groups (e.g., trans people and low‐income individuals).

Importantly, the impacts of structural stigma may not be felt the same for all members of a minoritized group. Postpartum health disparities, for example, may be pronounced for segments of the LGBTQ+ community, such as those who are bisexual, poor, of color, or gender nonconforming (Greenfield & Darwin, 2021; Januwalla et al., 2019). Indeed, the cost of seeking treatment may be higher for multiply marginalized persons. For instance, the risk of sharing postpartum mental health concerns with a provider may be greater for queer and low‐income individuals, who may be vulnerable to nonaffirming care (e.g., lack of understanding and insensitivity), or, possibly, particularly close scrutiny—including unwanted interference such as CPS referral (Kirubarajan et al., 2022; Polikoff, 2018).

The current study, then, focuses on a sample of mostly bisexual women partnered with men—a group identified as at risk for postpartum mental health disorders in particular, as well as mental health challenges in early parenthood more generally (Flanders et al., 2016; Goldberg et al., 2020), many of whom were also young (i.e., < 25 years old) and low income (i.e., 25% were below the federal poverty level for a family of three). Our sample also contains a significant minority of nonbinary birthing parents (i.e., 17%), who constitute an understudied but at‐risk group during the perinatal period, amidst the heteronormativity and cisnormativity embedded in the health care systems governing pregnancy, birth, and beyond (Besse et al., 2020). The goal was to understand these individuals' mental health experiences and concerns, and perceived barriers to help‐seeking, with a special focus on how concerns about seeking help might intersect with concerns about child welfare involvement amidst their multiply marginalized statuses.

## METHOD

Participants (*N* = 99)—all members of the LGBTQ+ community who were AFAB—were recruited via Prolific, an online recruitment platform that uses specialized targeting techniques to share surveys with pre‐registered respondents. Respondents were invited to participate in March–June 2023 if they (a) were the parent of at least one biological child born between 2018 and 2023, and (b) identified as LGBTQ. All respondents were rigorously prescreened by Prolific, which requires all individuals to undergo an identity check to ensure they are valid participants. The survey, which included closed‐ and open‐ended questions, was hosted on the online platform Qualtrics, and took an average of 36.7 min to complete. It was approved by Clark University's institutional review board.

Participants were told that the anonymous survey focused on LGBTQ+ parents' early parenting and reproductive experiences. Specifically, they were told that it included questions about family building, pregnancy, birth, new parenthood, mental health, social support, and reactions to recently passed legislation (e.g., overturning of *Roe v*. *Wade*).

We sought out a sample of individuals who were marginalized based on their sexuality and/or gender identity amid assumptions that these individuals might feel especially vulnerable with respect to both attacks on reproductive rights and attacks on LGBTQ+ people, both of which are advancing across the US and tend to be concentrated in similar regions (Choi, 2023; Klein & Gruberg, 2023). In 2023, over 500 anti‐LGBTQ+ bills were introduced or passed (e.g., that ban gender‐affirming care, allow health care providers to discriminate based on religious beliefs, and prevent teachers from talking about LGBTQ+ identities; Choi, 2023). We sought to include all members of the LGBTQ+ community for the larger project, although, for this study, we limited our sample to queer parents who had given birth due to our interest in postpartum mental health, thus excluding 23 cis gay/bisexual men and one trans woman.

### Sample description

Our sample (*N =* 99) consisted of 81 cis women (81.8%), 17 nonbinary AFAB individuals (17.1%), and one trans man (1%). In terms of sexual orientation, all participants identified within the queer (or LBQ+) umbrella. Most were bisexual (*n* = 64; 65%) or pansexual (*n* = 17; 19%), with the remainder identifying as lesbian (*n* = 5), queer (*n* = 2), or something else (*n* = 11; e.g., asexual and lesbian; asexual and pansexual; demisexual).

Among cis participants, 59 (60.8%) were bisexual, queer, or pansexual (BQ+) women partnered with cis men, 10 (10.3%) were unpartnered/single lesbian, bisexual, queer, or pansexual (LBQ+) women, 6 (6.1%) were LBQ+ women partnered with cis women, 4 (4.1%) were LBQ+ women partnered with nonbinary individuals, and 2 (2.1%) were LBQ+ women partnered with trans women. Among nonbinary participants, 10 (10.3%) were LBQ+ with cis men partners, 3 (3.1%) were LBQ+ with nonbinary partners, 3 (3.1%) were LBQ+ with trans women partners, and 1 (1.0%) was pansexual and unpartnered. The single trans man participant was bisexual and partnered with a cis man (1.0%).

Table 1 contains demographic characteristics of the sample including socioeconomic status, race, age, and U.S. region. Of note is that more than half reported a household income of under $50 K, and over a quarter were below the federal poverty level (HealthCare.Gov, 2023). Participants lived in 34 states, with greatest concentrations in TX (11), OH (8), NC (7), PA (6), FL (6), MI (6), and IL (5). Most lived in the South (42.9%) and Midwest (27.6%).

**TABLE 1:** Demographic characteristics of participants.

	Total (*n*)	Percentage (%)
Race and ethnicity		
White only	77	77.8%
Black	5	5.1%
Hispanic/Latinx	6	6.1%
American Indian/Alaska native (AI/AN)	1	1%
AI/AN and White	3	3%
AI/AN and Latinx	2	2%
Black and Asian	1	1%
Black and White	1	1%
AI/AN, Black, and White	1	1%
Latinx and White	1	1%
“Multiracial”	1	1%
Household income (HI)		
<$12.5 K^a^	7^a^	7.1%
$12.5 K–$25 K^a^	19^a^	19.2%
$25,001–$50 K	27	27.3%
$50,001–$75 K	22	22.2%
$75,001–$100 K	12	12.1%
>$100 K	12	12.1%
Self‐reported SES		
Lower class	18	18.2%
Working class	42	42.4%
Lower middle class	20	20.2%
Middle class	14	14.1%
Upper middle class	5	5.1%
Employment status		
Full‐time	41	41.4%
Part‐time	13	13.1%
Homemaker	38	38.4%
Unemployed	7	7.1%
Age^b^		
19–25	23	23.7%
26–30	36	36.4%
31–35	25	25.3%
36–40	12	12.1%
41–45	1	1.0%
Region of residence		
South	42	42.4%
Midwest	27	27.3%
Northeast	17	17.2%
West Coast	12	12.1%

^a^ Indicates participants who reported an income at or below the federal poverty level of $25 K for a family of 3; thus, over a quarter (*n* = 26, 26.2%) met this threshold (HealthCare.Gov, 2023).

^b^ Two participants were missing age data.

More than two‐thirds of participants had one child (*n* = 66, 66.7%); almost one‐quarter (*n* = 24, 24.2%) had two children, four participants (4.0%) had three children, and five participants (5.1%) had four children. Eighty‐eight (88.9%) had at least one child <4 years of age. Namely, 21 (21.2%) had one child <1 year, 22 (22.2%) had one child between 12 and 23 months, 19 (19.2%) had at least one child 24–35 months (18 had one child, one had two), 26 (26.3%) had at least one child 36–47 months (25 had one child, one had two), and 46 (46.5%) had at least one child ≥4 years (39 had one child, five had two, one had three, and one had four). Six (6.1%) were currently pregnant. Most (*n* = 88, 88.9%) had at least one child via intercourse with their partner; four (4.0%) had at least one child via intercourse with someone else. Three (3.0%) used intrauterine insemination to become pregnant, two (2.0%) used in vitro fertilization to become pregnant, five (5.1%) stopped hormones to become pregnant, and three (3.0%) took hormones to become pregnant. Six (6.1%) identified other ways they had become parents (e.g., pregnancy due to sexual assault).

Regarding pregnancy and birth history, 34 (34.3%) reported pregnancy loss, 44 (44.4%) reported pregnancy complications, 12 (12.1%) experienced gender dysphoria while pregnant, and 49 (49.5%) reported traumatic birth experiences. Fifteen (15.2%) reported a past abortion.

### Measures

The closed‐ and open‐ended questions in the survey were informed by the authors' knowledge of the relevant literatures, including their limitations, as well as our own prior research on related topics (Flanders et al., 2016; Goldberg, 2023; Goldberg et al., 2024). In developing the survey, the first author also consulted with several colleagues with expertise in queer communities and (a) poverty, (b) the child welfare system, and (c) parenting. The survey was proofed for functionality and ease of use by several graduate students in clinical psychology.

#### Closed‐ended questions

In addition to demographic items assessing gender, sexual orientation, age, race, income, partnership status, employment status, location, children, and parenthood route, we asked a variety of closed‐ended questions. Specifically, we asked about mental health conditions in the first postpartum year (i.e., depression, anxiety, bipolar disorder, OCD, or something else), and whether these were self‐ or provider diagnosed. We also asked how participants' mental health changed across the first year of parenthood: *significantly declined*, *somewhat declined*, *no change*, *somewhat improved*, *significantly improved*. We asked if participants had sought help from a therapist/other providers; if they experienced fears or worries related to seeking help; and, if so, to indicate the sources of those fears from a preset list (namely: fear of being judged by providers; fear of being deemed ‘unfit’; fear of the child welfare system/outside intervention; feeling that they should ‘suck it up’/be okay on their own; worries about how family/friends might view them; worries about homophobia/transphobia by the medical system; worries about racism by the medical system; other worries).

#### Open‐ended questions

Participants were asked several open‐ended questions: First, they were asked to elaborate on changes in their mental health over the first year of parenthood (“Please elaborate on [changes endorsed above]”). Second, they were asked to elaborate on their fears related to seeking help (“Please expand on [endorsed fears or worries]”). Third, they were asked, “If you sought out services (e.g., therapy) despite fears and/or worries, what pushed you to do so?” In our analysis, we also reviewed their answers to other open‐ended questions related to health care and parenting, namely: “Please share any other relevant experiences as an LGBTQ+ parent living and parenting in 2023”; “What guidance, support, and advice do you wish healthcare professionals gave you after you gave birth?”; and “How were your postpartum health care experiences inclusive or not inclusive of you as an LGBTQ+ person?”

### Data analysis

The current study is a mixed‐method study. Thus, we drew from both quantitative and qualitative data to inform our limited understanding of the unique challenges that multiply marginalized queer parents experience during early parenthood.

#### Quantitative analysis

Basic descriptive statistics were calculated for the sample. Of particular interest was whether, within our sample of queer parents, parents of color, parents whose income was below the poverty line, very young parents (25 and younger), single parents, and TNB parents, reported more fear of child welfare involvement than White parents, more financially secure parents, older parents, partnered parents, and cis female parents, respectively.

#### Qualitative analysis

Responses to the open‐ended survey portions ranged from one sentence to half a page of text, with most participants providing responses of 2–5 sentences. The first author used thematic analysis (Braun & Clarke, 2006, 2013) to examine responses from open‐ended questions.

The analysis focused on participants' reflections on their mental health, help‐seeking, and fears. The analysis was informed by prior literature and a structural stigma framework. The first author initially read all open‐ended responses to gain familiarity with the data, including overarching themes in responses. She made note of, and bracketed, her own experiences and preconceptions to facilitate a curious and open stance in relation to the data. Then, responses were annotated: via line‐by‐line coding, she labeled phrases relevant to the primary domains of interest (e.g., reasons for not seeking treatment; factors that pushed them to seek treatment). These codes were abstracted under larger categories and subcategories, which were positioned in relation to each other, such that connective links were established (e.g., links among mental health challenges, fear of stigma, the role of marginalized statuses) to meaningfully describe experiences as queer new parents. A tentative scheme was produced and reapplied to the data, such that all data were recoded according to the scheme. Themes were analyzed for the full sample and by key demographics such as sexual orientation and race. The second author served as an auditor and provided critical input at various stages of coding, as detailed below.

##### Trustworthiness

To enhance trustworthiness in study preparation and data collection, we pursued a data collection strategy (i.e., an online survey) that we believed would result in high‐quality and contextually valid data (Elo et al., 2014; Lincoln & Guba, 1985). We also posed both open‐ and closed‐ended questions in an effort to obtain multiple forms of data that would lend themselves to a deeper, richer understanding of the phenomena of interest (Morrow, 2005).

To enhance trustworthiness in the data analysis process, we as a research team sought to maintain reflexivity through open discussion of our assumptions and positionality throughout the process of examining, organizing, and interpreting the data (Morrow, 2005). To further enhance credibility of the analysis, the second author reviewed several versions of the coding scheme, providing input on each iteration and collaboratively examining the fit between the data and the emerging themes (Goldberg & Allen, 2015). Upon review of the final coding scheme, the second author made several suggestions for reorganization, and changes were integrated accordingly into the final thematic structure. After reaching the final thematic structure, the authors noted the absence of any new concepts, codes, or themes, indicating that data saturation had been reached (Lincoln & Guba, 1985). Finally, the authors selected meaningful and appropriate quotes from participants to include in the paper to illustrate key concepts (Morrow, 2005).

## RESULTS

### Mental health conditions, first postpartum year

Most participants (*n* = 88; 88.9%) described at least one mental health issue in their first year postpartum. See Table 2 for details regarding the incidence of each diagnosed mental health issue, and whether it was diagnosed by a provider or self‐diagnosed.

**TABLE 2:** Participant mental health experiences and diagnoses in the first year postpartum.

	Total	Provider diagnosed	Self‐diagnosed
Any mental health issue	88 (88.9%)	‐	‐
Major depressive disorder	64 (64.6%)	47 (73.4%)	17 (26.6%)
Anxiety disorder	64 (64.6%)	44 (68.8%)	20 (31.3%)
Obsessive–compulsive disorder	13 (13.1%)	6 (46.2%)	7 (53.8%)
Bipolar disorder	13 (13.1%)	6 (46.2%)	7 (53.8%)
Other diagnoses, including	19 (19.4%)	‐	‐
Posttraumatic stress disorder	9 (10.2%)	‐	‐

General mental health status^a^				
Declined considerably	Declined somewhat	No change	Improved somewhat	Improved considerably
41 (41.4%)	32 (32.3%)	9 (9.1%)	8 (8.1%)	5 (5.1%)

^a^ Four participants missing data on general mental health.

When asked to elaborate on their experiences of and changes in mental health across the first year postpartum, some spoke to the subjective experience of postpartum depression, anxiety, OCD, and paranoia, as well as factors they perceived as contributing to or underlying their distress. Frequently noted contributors were *isolation and lack of social support*. Some asserted that they were estranged from family, some described having few friends, and some noted the absence of partners (or had partners who were physically absent, e.g., deployed; or emotionally absent, e.g., they were “not helpful”). Kara, a White cis bisexual single woman in Ohio, said, “I have no partner. There is little to no support for single parents, men and women, who are just above the poverty line. We are drowning, just a little slower than everyone else.” Dani, a White nonbinary asexual married parent in North Carolina, had “really bad PPD” after the birth of their child, yet “no one really listened to me or thought anything was really wrong. I could hardly get out of bed for a long time [but] I had no support, not even with household chores.”

Some noted tensions in their *partner relationships* that exacerbated their mental health challenges. Sol, a biracial (White/American Indian) cis bisexual married woman in Kansas, said, “I had a really awful time with postpartum depression. Because we could not afford child care, I had to stay home full‐time while my partner worked 60–75 hours a week. We were both exhausted, financially strapped, and fell out of good communication habits.”

In a few cases, participants ended their relationships with partners, which exacerbated their isolation, financial challenges, and mental health challenges. Chloe, a White cis bisexual single woman in Michigan, shared, “Within the first year, my partner left. I was depressed and suicidal. Over time, I slowly gained more support and fought my way out of mental hell.” Devon, a White nonbinary single lesbian parent in Washington, said:

Being responsible for a baby meant I could no longer endure the abusive relationship I had. I had already made multiple attempts to leave. If I did [stay], I would have not been a good parent … I broke it off despite having to remain living together for the baby … At the same time I lost all my friends and had a very tense relationship with my family of origin.

Some participants highlighted *financial challenges* as a stressor. They noted how their sense of financial security became even more precarious once they were parents and the reality of caring for a human set in (“Babies cost money, plus hospital bills”; “Children are expensive”).

Some nonbinary participants highlighted their *gender dysphoria* during pregnancy or postpartum as impacting their mental health, describing the “discomfort” and “distress” they experienced related to their pregnant bellies or chest growth. Mel, a White nonbinary asexual married parent in North Carolina, said, “I had a traumatic birth and struggled with PPD and I had gender dysphoria during and after pregnancy because there wasn't much I could do about my gender presentation.” Devon, a White nonbinary single lesbian parent in Washington, said:

I experienced pre and postpartum depression and anxiety that caused suicidal ideation. During pregnancy I couldn't look at myself as it made my “feminine” features more prominent and for about a year postpartum I was very emotionally distraught about the presence of my vagina.

Several participants said that the *pregnancy was unplanned*, which impacted their distress. Julia, a biracial (White/American Indian) cis bisexual partnered woman in Illinois, said, “[I was] depressed with how the pregnancy came to be. I really just tried to shove my feelings down and clean and work as much as I could until I couldn't anymore.” Finally, *traumatic birth experiences* were named by several participants as impacting their postpartum mental well‐being. Kate, a White cis bisexual married woman in Texas, attributed her postpartum depression and anxiety in part to her “unsupportive midwife team and hospital birth provider who did not respect my wishes, resulting in a sense of loss of self and loss of power which was traumatic, and…irreversible choices made for my baby without my consent.”

### Fears and worries about seeking help: Quantitative

Two‐thirds of participants with mental health problems in the postpartum period (56 of 88; 63.6%) endorsed having sought help for these difficulties. Many of those who did not seek help, and some who eventually did, endorsed worries related to seeking help, specifically: fear of being judged by providers (*n* = 46, 46.9%); fear of being deemed “unfit” (*n* = 57, 58.2%); fear of the child welfare system (*n* = 37, 37.8%); feeling that they should “suck it up”/be okay on their own (*n* = 54, 55.1%); worry about family/friends might view them (*n* = 22, 22.4%); worry about homophobia/transphobia by the medical system (*n* = 4, 4.15%); and worry about racism by the medical system (*n* = 3; 3.1%). Three named other worries (feeling it would not help; worry about being able to afford help). Of note is that of the four participants worried about homophobia or transphobia, three were nonbinary, and all were pansexual. Of the three worried about racism, two were Black and one was Hispanic.

Given our particular interest in fears surrounding child welfare involvement, and whether they might be more pronounced in vulnerable groups, we explored via a series of chi‐squares whether participants who were of color (vs. White), had incomes below the poverty line (vs. more financially stable), were in the youngest age category (25 and under, vs. those over 25), single (vs. married/partnered), and TNB (vs. cis), were more likely to fear CPS. Participants with incomes below the poverty line were more likely than those with higher incomes to endorse this fear, *χ* ^*2*^ (1, 98) = 6.874, *p* = 0.010, and those age 25 and younger were more likely than older participants to endorse this fear, *χ* ^*2*^ (1, 98) = 4.02, *p* = 0.041. Parents of color were not more likely than White parents to fear CPS, *χ* ^*2*^ (1, 98) = 0.183, *p* = 0.791; nor were single parents more likely than partnered parents (*χ* ^*2*^ (1, 98) = 1.41, *p* = 0.286) or TNB parents more likely than cis parents (*χ* ^*2*^ (1, 98) = 0.296, *p* = 0.618) to endorse this fear.

#### Fears and worries about seeking help: Qualitative

**FIGURE 1:**
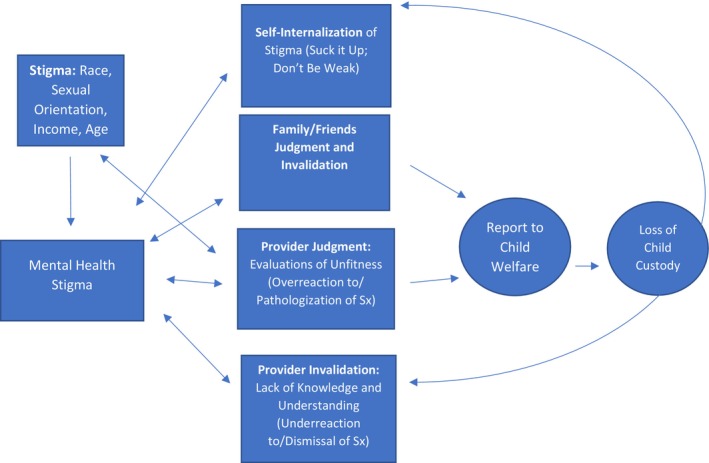
Relationships among stigmatized statuses, mental health stigma, fears of help‐seeking, and fears of child welfare involvement.

When asked to elaborate on their fears and worries about seeking help for their mental health issues in an open‐ended manner, participants' narratives nuanced their responses to the quantitative items. They highlighted awareness of mental health stigma, both internalized (I should be fine) and externalized (others will judge me), and fear of negative treatment by providers, including not taking their problems seriously, and judging them as mentally unwell or a bad parent, which could prompt child welfare involvement. Fear of racism, homophobia, and transphobia intersected with concerns about negative treatment by providers or CPS. Similar to fears about providers, some voiced fears about how family/friends might not take their distress seriously. See Figure 1 for a visual depiction of how these themes intersect.

##### Mental health stigma

Some participants spoke to the salience of mental health stigma in their reluctance to seek help. They voiced an awareness of negative associations related to mental illness, recognizing that people who struggled with their mental health could be judged as “crazy,” and incapable of holding down a job or parenting, which discouraged help‐seeking. Leah, a White cis pansexual partnered woman in Rhode Island, said, “I think the idea of starting therapy is so terrifying for so many people simply because it isn't talked about and people still view it as something bad.” Chace, a White nonbinary pansexual partnered parent in Wisconsin, described their “generalized fear of getting help due to the idea, instilled by my family, that psychiatric help would disqualify me from certain jobs or cause intervention by CPS.” In several cases, participants described mental health stigma specifically related to the perinatal period, noting how (a) new parents are generally expected to embody an attitude of joy and well‐being, and (b) voicing negativity or sadness seemed to carry particular risk for harsh judgment during this period. Danya, a White cis bisexual married woman in Texas, said, “Postpartum depression is so scary. I was so worried about how people would react if they knew I wasn't feeling ‘the happiest I've ever been.’”

###### Internalized mental health stigma

The belief that their mental health struggles made them incompetent or bad parent was internalized by some parents. Bella, a White cis bisexual married woman in Ohio, who struggled with postpartum depression, shared, “I really felt as if I was a bad mom, and I worried that I would say or do the wrong thing.” Maya, a Hispanic cis pansexual married mother in Colorado: “I was worried my mental health meant I was a bad mom, [and] that I couldn't take care of my kids as well as others.” The belief that one should “suck it up” was prominent for several participants, and kept them from sharing their mental health struggles with others. Ella, a multiracial cis asexual married woman in Alabama, noted, “Self‐reliance is prized in our society, and I generally stick to this with everything.”

###### Mental health stigma among family

Several participants described how mental health stigma and lack of awareness held by family members had exacerbated their own negative feelings about their mental health, and fear of seeking help. They had attempted to talk about their mental health challenges with family, only to meet invalidation or judgment. Tillie, a White cis bisexual married woman in Virginia, shared: “I knew postpartum depression existed but when I reached out to my mom she was very negative about it and told me she couldn't understand why I was having these issues.” This response discouraged her from seeking help. A quote by Caryn, a White cis pansexual married woman in Pennsylvania, revealed how internalized stigma was exacerbated by family members' responses, which discouraged help or support seeking:

I just felt that I was being a bad mother. My child wouldn't latch, I couldn't produce any milk, I have a lot of built up childhood trauma and guilt, my family were no help other than to say that only crazy sick people see therapists and take meds and I shouldn't because [of] what other people think of me and the family.

##### Fear of Providers' judgment as “unfit”, prompting child welfare intervention

Many participants spoke about their fears of being labeled or marked as a “bad parent” by health care providers because of their mental health challenges (anxiety, depression, panic attacks, intrusive thoughts). Maddie, a White cis bisexual married woman in Iowa, said, “I was afraid that they would think I wasn't a good enough mother.” LJ, a multiracial married participant of undefined gender and sexual orientation in New York, said, “I worried about how I would be perceived and if [they] would think my mothering was less than competent.” Many spoke to how they feared that this judgment—i.e., them being deemed an unfit parent—might trigger child welfare involvement, and, ultimately, loss of a child to CPS—or, in a few cases, to a vindictive ex‐partner (“I think my ex … will use my mental health against me”). Callie, a White cis bisexual single woman in Georgia, said: “My biggest fear about getting mental health help is that my child will be taken away from me. I have heard too many stories of women coming forward about their mental health issues and their children being taken by CPS.” Cindy, a Hispanic cis bisexual married mother in Texas who dealt with postpartum anxiety, said, “I have an immense fear of health care providers knowing about my worries and [having my] child taken away for any reason.” Participants were aware of their vulnerability as new parents seeking help. Joyce, a White cis bisexual partnered woman in Florida, said, “Saying ‘I'm not okay’ when you're a new parent is extremely [risky] … because things like involuntary holds and CPS exist.”

Several participants identified additional “marks” on their record that added to their mistrust of CPS. Specifically, a few parents had criminal records and were worried about this “coming into play” if they reached out for mental health support. Parenting practices (e.g., gender‐neutral parenting, co‐sleeping) were also noted as potential “red flags” that could cause a provider or someone else to “call CPS on us.” Danya, a White cis bisexual married woman in Texas, said:

I was nervous about some of the choices we made as parents. We decided to co‐sleep, and we don't do baths every single day. I know that probably isn't child services related, but I was so scared someone would call me in and try to take my baby away…I do have a criminal history and it's not a secret, so I was worried that might come into play as well.

Prior negative experiences with CPS, and associated mistrust of the system, were identified by some participants as informing their current fears. For some, their experiences with CPS occurred when they were children (“I was a poor foster kid”) while others had experiences as adults (“due to gender neutral parenting ideas, I did have CPS involvement; they closed the case but there's still a mark in my record”). Such experiences were uniformly negative and discouraged participants from seeking help for fear of reengaging these systems. Charlie, a White nonbinary asexual married parent in Michigan, said: “After birth, I had CPS involvement due to money, and my mental health being brought up to them by someone.” Noting that they were “misdiagnosed for many years as bipolar,” that was “all the providers saw. They didn't want to see postpartum depression or anxiety. I was just crazy and unfit.”

These participants, then, grounded their fears of help‐seeking related to mental health in their knowledge of “how the system works” rendering them fearful of social service oversight. Lena, a Hispanic cis bisexual partnered woman living in California, shared:

The way these agencies function under the guise of “helping” was a huge barrier to seeking help for me. I know what they are capable of and I know I can't trust them. Any potential benefits from mental health treatment I could receive are greatly overshadowed by the trauma and heartache that I would impose on my loved ones and myself if a provider interpreted my situation as deserving of a CPS referral.

###### The role of marginalized identities

Some parents spoke to specific identities, which, in intersection with their mental health, might invite greater scrutiny or bias by health providers, potentially leading to CPS referral. Specifically, and somewhat consistent with our finding that lower income respondents identified fear of CPS as a barrier to mental health seeking, some participants named their *income* as a factor in their vulnerability, such that being poor, combined with having mental health issues, rendered them vulnerable to evaluations of “bad motherhood” or unfitness (“I was scared that they would judge me because I'm low‐income and have anxiety”; “DCS is always coming for low‐income families”). And, consistent with our finding that young participants were more likely to endorse fear of CPS as a reason for not seeking treatment, some parents spoke to their *young age* as a factor that could put them at risk for negative judgments by health providers. Reese, a White nonbinary single parent who declined to put a label on their sexual orientation, and who resided in Washington, said:

I have received a lot of judgmental comments from providers and was even told my best bet as a 19‐year‐old was to have a late term abortion … I felt these people felt I was entirely unfit in some irredeemable manner and my PPD [postpartum depression] would only empower them to take my baby or hospitalize me against my will.

Several participants, all of whom were TNB and/or their partners were TNB, emphasized their *queerness or gender identity/presentation*, such that they believed that their mental health challenges or seeking help for such challenges could invite intensified scrutiny. Elle, a White nonbinary pansexual participant in New Jersey, who was married to a trans woman, said, “I live in constant fear that someone is going to call child services on us because of our identity.” Sage, a White nonbinary pansexual married parent, partnered with a cis man in Arkansas, shared, “I don't want people to take my child away because we're queer. It's a real fear.”

Several participants, all Black, spoke to *race* in intersection with their mental health in rendering them at risk for evaluations of parental unfitness. Speaking to her identity as a Black queer woman, Gwen, who was married and lived in Florida, said, “I was greatly worried that I would not be given the same care due to race, and that my struggles would make it more likely that the state would intervene.” She added, “The area we live in is known for taking Black children for minor or non‐existent reasons.” Cara, a Black nonbinary “feminine presenting” pansexual parent who was separated, single, and living in Florida, “Since I am a Black woman, I was mostly scared of being seen as unfit for voicing my feelings and concerns.”

A few participants spoke to their *single parent status*. Kelly, a White cis bisexual single mother in Missouri, noted, “A history of self‐harm is [especially] damaging to single mothers, who are viewed [negatively].”

##### Fear of dismissal and invalidation by health care providers

Some participants, in sharing why they did not seek help or delayed seeking help for their mental health challenges, spoke to fears of being undermined or invalidated by providers, which were often grounded in prior experiences. Thus, they held back on sharing their concerns less because of a fear of being explicitly judged or flagged as “unfit” (i.e., having their mental health issues “overreacted” to, possibly leading to CPS referral) and more because they did not expect to be taken seriously or they felt helpless amid prior experiences. Some had raised their concerns with providers and felt “brushed off”, which they often related to how women and AFAB people are treated by health providers in general—that is, their distress is not believed or is minimized. Notably, most of these parents lived in “red” states in the South and Midwest. Gavin, a White trans bisexual man in Florida, said, “Constantly being told that the way I felt was just ‘typical’ for pregnancy and postpartum did not ease my unrest and left me to just get worse till I felt that speaking up was completely useless.” Mika, an American Indian cis pansexual married woman in Oklahoma, said, “Doctors here don't take women seriously. If you go in wanting to talk about anxiety or depression, you're treated like you are just on your period. If you push further, you're considered hysterical.” Several participants spoke about how prior negative encounters had informed their mistrust of providers. KK, a White nonbinary asexual married participant in North Carolina, shared, “I felt like I would be judged for having mental health issues or that they wouldn't believe me about them. I tried to get help before and it seemed like they just brushed me off.”

A few participants shared how their experience of having their mental health issues deflected or ignored intersected with the erasure they experienced as LGBTQ+ people. Blair, a White nonbinary pansexual married parent in Maryland, said: “I wasn't given any postpartum check‐ups except for those yes or no question sheets that do nothing but make you feel like you're making it up. There was no mention of LGBTQIA+ in any paperwork … It was ignored.” A few shared their uncertainty about whether their sexual orientation or gender identity was a factor in providers' dismissive treatment of them. Lee, a nonbinary asexual partnered lesbian in Michigan, reflected, “I'm not sure if it's because of my gender nonconformity or not. Is it because I'm a female, because I don't look feminine, or because I'm poor? I [don't] know.”

#### Seeking help: What prompted new parents to seek out care/treatment

Two‐thirds of participants with mental health issues (*n* = 56, 63.6% of 88) endorsed having reached out for help. When asked what led them to seek support or help from a provider, many noted that they *wanted to be the best parent they could*, for themselves and their children. Tre, a nonbinary pansexual married parent in Wisconsin, said, “I need to be healthy, begin healing from my trauma, and be well‐rounded emotionally to raise emotionally healthy and non‐wounded children.” Rosie, a White cis bisexual married woman in Michigan, said, “I needed to get help to be a better parent. My kids were more important than what people thought of me.”

Other participants were prompted by the fact that their mental health issues had *reached a* “*tipping point*,” where they were “no longer functioning” or were suicidal (“I had thoughts of suicide”; “I was doing really bad mentally”). Hope, a White cis bisexual single mother in Ohio, said, “My mental illness was not letting me properly be there for my child.” Sol, a biracial (White/American Indian) cis bisexual married woman in Kansas, said, “It was either seek help or kill myself. I could not bring myself to invite that type of trauma onto my family.”

In some cases, participants' *partners pushed or begged them to seek help*, with several participants noting that their mental health difficulties had seriously impacted not only their parenting but also their intimate relationship (“My husband told me I should, for my children”; “My partner begged me to get help”). And, a few participants simply said that the benefits of seeking out therapy or medication, such as a potential reduction in symptoms, outweighed the possible risks, such as having their child taken away or others seeing them as incompetent.

Some parents said that they wished that it was easier to get treatment. Specifically, they wished that postpartum mental health support was offered to them after giving birth—which in some cases was several years earlier—highlighting how normalizing such care and making it accessible could have saved them significant distress. Mel, a White nonbinary asexual married parent in North Carolina, said: “I wish I had support at all after I gave birth. My doctors seemed to quit caring about me after I left the hospital.” Cleo, a cis White bisexual married woman in Texas, said, “I wish someone had … asked me how I was. I felt like no one gave a care once I had my kid. I wasn't important.” Lisa, a White cis bisexual single woman in Texas, revealing how completely inconsequential she felt after giving birth, said, “No one asked, and I didn't tell. I'm just a single mom.” Those who received resources felt that they were not helpful or meaningful, in part because they were not accompanied by provider follow‐up. Devon, a White nonbinary single lesbian parent in Washington, said, “I wish I had been given more options for mental health care. I was given one pamphlet about PPD, and told my only choice was to go inpatient which was not financially feasible. This left me alone and afraid to ask for help.” Dani, a White nonbinary asexual married parent in North Carolina, shared, “I got a single brochure about PPD, and it wasn't that helpful, even when I tried to talk to my doctor about it.”

## DISCUSSION

This study makes several contributions to the literature on queer parents' well‐being in early parenthood. First, we documented high rates of postpartum mental health challenges for queer birthing parents and factors that may help to explain distress, several of which are unique to this population. Second, we describe how structural stigma creates barriers to help‐seeking for queer new parents—identifying ways that child welfare systems in particular can be perceived as forces of harm by struggling, multiply marginalized parents. Third, we note factors that may help queer parents to overcome barriers to help‐seeking and access care despite structural stigma.

A notable finding is the high levels of postpartum mental health challenges, particularly mood and anxiety disorders, according to participants' retrospective self‐reports. The fact that more than three‐quarters of parents reported mental health challenges after birth (compared to 12%–35% of people overall; Neely et al., 2023) may, in part, be an artifact of our sampling procedure, in that our call for participants mentioned mental health and parenting. It may also reflect the fact that we sought new parents who were also LGBTQ+, a group that may be at higher risk for postpartum mental health problems (Kirubarajan et al., 2022). In one recent study, more than one‐third of sexual minority women scored in the clinical range for depression in the perinatal period (Marsland et al., 2022). Another recent study (Soled et al., 2024) found that lesbian, bisexual, and mostly heterosexual women were more likely to experience stress, depression, and antidepressant use than completely heterosexual women in the perinatal period. Further, our sample was largely made up of bisexual women partnered with men, a group that may experience unique mental health risks during the perinatal period (Goldberg et al., 2020).

In contextualizing their mental health, participants emphasized isolation, lack of partner support, financial issues, difficult birth experiences, and gender dysphoria. Some of these—such as isolation, partner issues, and financial issues—mirror prior study findings on factors that exacerbate postpartum mental health challenges in the general population (Neely et al., 2023) and LBQ+ women specifically (Ross & Goldberg, 2016). It may be that queer birthing parents experience such challenges at greater levels due to structural stigma and heterosexism. Indeed, queer people experience higher rates of poverty than heterosexual people (Badgett et al., 2019) and can experience social isolation from rejecting communities (Oswald et al., 2018) and familial rejection because of their LGBTQ+ status (Reczek & Bosley‐Smith, 2021)—although of note is that our participants did not specifically invoke their sexual orientation or gender identity in describing a lack of support; rather, they focused on the pain and isolation associated with it.

Some factors named by participants as impacting their postpartum mental health are unique to queer postpartum parents, including experiences of gender dysphoria throughout their pregnancies and the postpartum period. This finding underscores the particular vulnerability of an understudied group within the LGBTQ+ community: non‐cisgender individuals who give birth, whose embodied experience of pregnancy and parenthood are uniquely shaped by the clash between their gender identity and the cisnormative nature of health care and birthing environments (Besse et al., 2020). As nonbinary people (as opposed to trans men, on which most work in this area has focused; Besse et al., 2020; Greenfield & Darwin, 2021), they may be especially vulnerable to invisibility and erasure in the perinatal health context, in that nonbinary identities are less familiar to health providers (Goldberg et al., 2019).

While two‐thirds of participants sought help for mental health challenges, many delayed seeking help, and one‐third did not seek help. The potential for delayed treatment‐seeking is consistent with Soled et al. (2024), who found that bisexual and mostly heterosexual women were more likely than heterosexual women to wait until their perinatal mental health symptoms became severe to use antidepressants. Our participants named various factors that prevented or delayed seeking help, including fear of provider judgment (almost half), fear of being deemed “unfit” by providers and exposed to child welfare scrutiny (over half), and distrust of CPS (over one‐third). These fears are intersecting and overlapping, as shown in the qualitative data.

Our qualitative findings surfaced mental health stigma as a barrier to help‐seeking and disclosure. Participants demonstrated internalized mental health stigma: feeling that they should be able to manage their symptoms and improve on their own and/or that needing help was a sign of weakness or made them bad parents. They also worried that their friends and family might view them negatively or disapprove of them seeking help. Thus, our qualitative data highlighted the role of mental health stigma—internalized and externalized, by providers, family, and friends—as a salient consideration, echoing prior work on barriers to help‐seeking for postpartum mental health issues (Hadfield & Wittkowski, 2017; Hansotte et al., 2017).

Participants' narrative data further revealed that they worried that providers would judge them especially harshly and/or overreact to their difficulties (e.g., because of intersecting stigmas related to mental health, LGBTQ+ identity, poverty, and other social locations), leading to a referral to CPS. Participants explained how accessing services and sharing concerns would expose them to possible child welfare involvement—and they articulated their awareness that bias and stigma could increase the likelihood that they were referred to CPS and could lead to worse treatment and outcomes from CPS (e.g., child removal). Significantly, our quantitative analyses revealed that poor participants, and young participants, were especially likely to name the fear of CPS referral as a barrier to help‐seeking, echoing prior work suggesting that these and other subgroups of queer mothers may be especially vulnerable to stigma and CPS involvement (Harp & Oser, 2016; Kuri, 2020; Polikoff, 2018), and is consistent with economic disproportionalities in child welfare‐involved families (Fong, 2017, 2019).

Our data are also consistent with work by Johnson et al. (2023) documenting how, from the perspective of perinatal health care providers, stigma associated with perinatal mental health and fear of child care removal were viewed as key barriers to patient disclosure of mental health care challenges. Further, and notably, Forder et al. (2020) documented how women at greatest risk for perinatal mental health challenges were the least likely to be open about such challenges with providers, often due to fear of stigma and fear of child welfare involvement. It is reasonable that at‐risk groups may be less likely to disclose their mental health struggles because of perceptions of heightened risks and low rewards associated with disclosure. Notably, none of our participants saw possible child welfare involvement as a positive support or source of potential resources and services—and, some shared that prior child welfare system involvement (as a child or adult) influenced their negative opinion of the system and efforts to avoid contact with them.

Although participants who were single, of color, and/or TNB were not statistically more likely to fear CPS than their partnered, White, and cis counterparts, the qualitative responses highlighted how fear of provider mistreatment and CPS referral were especially nuanced for some TNB and Black participants. TNB participants recognized that their gender expression and/or queer identities rendered their parenting more visible and prone to scrutiny, especially in the current sociopolitical landscape, which is characterized by heightened hostility toward LGBTQ people and parents (Radis & Nadan, 2021). Likewise, Black parents are likely aware of the overrepresentation of Black families in the child welfare system (Cénat et al., 2021; Fong, 2017), and may also be sensitized to their unique risk for bias as Black queer parents (Harp & Oser, 2016). Members of both groups ultimately recognized their risks of CPS involvement and took measures to avoid such risks, pointing to how multiply marginalized queer parents are mindful of their intersectional risk with mental health stigma and identity‐based bias and may delay or avoid treatment to protect themselves and their families.

As participants wrestled with the decision to seek help for their mental health difficulties, some also experienced fears of invalidating experiences from a provider. Thus, these queer parents reported limited confidence that treatment providers could help them—perceptions that were sometimes grounded in prior negative interactions with health providers (Dennis & Chung‐Lee, 2006; Hadfield & Wittkowski, 2017), perhaps reflecting the larger impact of structural stigma in the health care system (Hatzenbuehler & Link, 2014). Still, over two‐thirds eventually faced or overcame such fears to access help, motivated largely by desire to be good parents, and sometimes fueled by the awareness that they were reaching a “breaking point” in their well‐being, or, by partners' pressure to seek help—revealing the potentially critical role of partner support in mental health treatment for new parents (Davey‐Rothwell et al., 2017).

### Implications

The individuals in our study are multiply marginalized, and have many reasons to mistrust and avoid mental health treatment and other interventions. We concur with Forder et al. (2020) in underscoring the need for providers to exhibit an open, nonjudgmental, and reassuring stance in relation to patients to reduce the stigma and fears that contribute to a reluctance to disclose. Further, our participants highlighted the importance of providers attending to parents' mental health proactively and providing resources for mental health care even when parents do not feel comfortable or safe voicing a particular need. Such efforts can increase patient–provider relationships and aid in appropriate treatment and referrals—which may enable parents to maintain custody of children, which will have positive, cost‐saving impacts on families. Indeed, child removal is related to worsened mental health issues for new mothers, and may contribute to intergenerational trauma (Kenny, 2018; Wall‐Wieler et al., 2018).

Many participants articulated fear of the child welfare system, seeing it as a threatening and biased entity with the power to coerce, control, and harm their families. Polikoff and Spinak (2021) argue that the child welfare system—or the family regulation system—causes damage to families, even when child removal is not the outcome of its involvement. This system, they assert, treats families as the source of the problems to be addressed, and “purports to address those problems through surveillance, intervention in family life, deep reliance on removing children, and providing services to families that rarely support their complex needs” (p. 433). We align with Polikoff and Spinak in recognizing that this system is not an effective substitute for investing in communities. Family practitioners can advocate for intentional development of housing, child care, mental health, and educational opportunities for all families, and engage in prevention and intervention efforts that address individual, relational, and contextual stressors, such as job loss, family communication challenges, and financial need (Kanter et al., 2021). Such efforts may help to improve family well‐being and reduce the likelihood of CPS involvement.

Our study findings identify intervention points for individual and family practitioners to better support queer birthing parents through their postpartum and early parenthood journey. First, there is a need for awareness of the impact of low‐income status and system distrust on mental health help‐seeking—and how intersections among income, race, and gender identity and sexual orientation may lead to particularly high levels of mistrust and avoidance associated with help‐seeking (Fong, 2019; Goldberg, 2023; Sødal et al., 2023). Practitioners must seek to build trust with vulnerable communities, and connect families with community resources that can help to decrease the likelihood of child welfare system referral or involvement. Notably, our findings also suggest that certain motivations for help‐seeking (such as the desire to be a good parent and partner pressure to seek help) may be important inflection points for intervention; indeed, family practitioners in health care settings should draw on these in their compassionate efforts to encourage and cultivate trust in high‐quality, affirming mental health treatment. Finally, our findings also underscore the need for increased training of providers, including therapists and nurses, on working with queer and TNB birthing people in the perinatal period and beyond. Practitioners who interface with this population must be alert to their unique needs and concerns, and the reality that they may carry with them past experiences with biased, insensitive providers.

### Limitations

Our study is limited in a number of ways. Indeed, both a strength and limitation of our sample is that a large number of our participants were bisexual cis women partnered with men. This is an important feature of our design, given that (a) this group may be at elevated risk for mental health concerns (including during the perinatal period), and (b) they are often invisible in studies of LGBTQ+ parents, especially childbearing individuals. However, we had few female‐partnered cis women in our sample, limiting our ability to document and illuminate this group's experience vis‐a‐vis mental health, help‐seeking, and child welfare system concerns. A second limitation is that we had too few participants in various racial/ethnic groups to meaningfully describe racialized intersections with other marginalized statuses. Indeed, in addition to Black mothers being at greater risk for child removal, American Indian/Alaska Native women are also at elevated risk—and may experience unique fears related to child welfare systems due to their history of mistreatment in the U.S. (Landers et al., 2023).

A third limitation is that we asked participants to indicate their income range, not their precise income, limiting our assessment of poverty level. Relatedly, in asking about barriers to help‐seeking, we did not explicitly inquire about financial challenges. Fourth, insomuch as we conducted an online mixed‐methods survey, we were not able to probe participants' responses to the degree that we would be able in an in‐depth survey. For example, research documents heightened fear of disclosure surrounding bisexual identity, isolation and lack of support, trauma, poverty, and intimate partner violence among bisexual people (National LGBT Cancer Network, 2024). It would be ideal to probe whether and how these issues intersect to impact mental health and fear of help‐seeking in this particular sample; future interview‐based methods can pursue this. Similarly, we documented themes of isolation and lack of support in relation to family, which undermined mental health and help‐seeking; however, it was unclear whether family frictions had increased since the birth of their children and/or in the current sociopolitical climate. Future work can explicitly probe for this. Fifth, we asked participants to report on their mental health retrospectively. Although all parents had young children, some had given birth several years prior, and their recollections may have been imperfect due to the passage of time.

## CONCLUSIONS

Queer parents, particularly those who are low‐income, racially minoritized, and/or have other intersecting minority statuses, are poorly represented in the literature—especially research on early parenthood, when parent mental health may be especially vulnerable and significantly impacts parenting and child well‐being. Building on a nascent literature that explores difficult topics among multiply marginalized and vulnerable queer parents (e.g., Harp & Oser, 2016; Januwalla et al., 2019; Kuri, 2020), we explored mental health and help‐seeking, with special attention to fear of child welfare system involvement, among queer new parents. Our findings generate much‐needed insights into the lived experiences of queer parents living at the margins, who are rarely centered in research on queer parents (Goldberg, 2023; Lavender‐Stott & Tasker, 2020). Future work should build on our findings to provide more than a snapshot into their lives, seeking to understand how their experiences of identity, risk, resilience, parenting, and well‐being intersect and unfold over time, particularly in the current sociopolitical climate, where LGBTQ+ rights are debated and LGBTQ+ lives are under threat (Washington, 2022).

## FUNDING INFORMATION

This research was funded by Jan and Larry Landry Endowed Chair funds, awarded to the first author [Correction added on 05 August 2024, after first online publication: Funding Information section has been added in this version.].
